# Supplementary material for: HIVprotI: an integrated web based platform for prediction and design of HIV proteins inhibitors
Source: J Cheminform. 2018 Mar 9;10:12. doi: 10.1186/s13321-018-0266-y (PMC5845081; doi:10.1186/s13321-018-0266-y)
Supplement: Supplementary file 2 — Additional file 2. Source code of HIVProtI web server. [file 13321_2018_266_MOESM2_ESM.zip › HIVprotI_Source-code/hivprotI/weka-3-6-6/doc/weka/attributeSelection/RankedOutputSearch.html]

RankedOutputSearch


---


|  |  |  |
| --- | --- | --- |
| **Overview** \| **Package** \| **Class** \| **Tree** \| **Deprecated** \| **Index** \| **Help** \| **Weka's home** | |  |
| **PREV CLASS**   **NEXT CLASS** | **FRAMES**    **NO FRAMES**     **All Classes** |  |
| SUMMARY: NESTED \| FIELD \| CONSTR \| METHOD | DETAIL: FIELD \| CONSTR \| METHOD |  |


---


## weka.attributeSelection Interface RankedOutputSearch

**All Known Implementing Classes:**: GreedyStepwise, RaceSearch, Ranker

---

``` public interface RankedOutputSearch ```

Interface for search methods capable of producing a
ranked list of attributes.

**Version:**
:   $Revision: 1.11 $

**Author:**
:   Mark Hall (mhall@cs.waikato.ac.nz)

---

| **Method Summary** | |
| --- | --- |
| `int` | `getCalculatedNumToSelect()`             Gets the calculated number of attributes to retain. |
| `boolean` | `getGenerateRanking()`             Gets whether the user has opted to see a ranked list of attributes rather than the normal result of the search |
| `int` | `getNumToSelect()`             Gets the user specified number of attributes to be retained. |
| `double` | `getThreshold()`             Gets the threshold by which attributes can be discarded. |
| `double[][]` | `rankedAttributes()`             Returns a X by 2 list of attribute indexes and corresponding evaluations from best (highest) to worst. |
| `void` | `setGenerateRanking(boolean doRanking)`             Sets whether or not ranking is to be performed. |
| `void` | `setNumToSelect(int numToSelect)`             Specify the number of attributes to select from the ranked list. |
| `void` | `setThreshold(double threshold)`             Sets a threshold by which attributes can be discarded from the ranking. |

**Method Detail**

### rankedAttributes

```
double[][] rankedAttributes()
                            throws java.lang.Exception
```

:   Returns a X by 2 list of attribute indexes and corresponding
    evaluations from best (highest) to worst.

    :   **Returns:**: the ranked list of attribute indexes in an array of ints **Throws:**: `java.lang.Exception` - if the ranking can't be produced

---


### setThreshold

```
void setThreshold(double threshold)
```

:   Sets a threshold by which attributes can be discarded from the
    ranking. This threshold is used by the AttributeSelection module
    which does the actual discarding of attributes---the implementer
    of this method needs only to provide a variable in which to store the
    supplied threshold. -Double.MAX\_VALUE is reserved to mean no threshold,
    ie, retain all attributes.

    :   **Parameters:**: `threshold` - the threshold.

---


### getThreshold

```
double getThreshold()
```

:   Gets the threshold by which attributes can be discarded. Discarding
    of attributes is done by the AttributeSelection module using the
    threshold returned by this method.

    :   **Returns:**: a threshold by which to discard attributes

---


### setNumToSelect

```
void setNumToSelect(int numToSelect)
```

:   Specify the number of attributes to select from the ranked list. < 0
    indicates that all attributes are to be retained. NumToSelect has
    precedence over threshold, ie. if there is a non -1 value for NumToSelect
    then this will take precedence over any threshold value.

    :   **Parameters:**: `numToSelect` - the number of attributes to retain

---


### getNumToSelect

```
int getNumToSelect()
```

:   Gets the user specified number of attributes to be retained.

    :   **Returns:**: the number of attributes to retain

---


### getCalculatedNumToSelect

```
int getCalculatedNumToSelect()
```

:   Gets the calculated number of attributes to retain. This is the
    actual number of attributes to retain. This is the same as
    getNumToSelect if the user specifies a number which is not less
    than zero. Otherwise it should be the number of attributes in the
    (potentially transformed) data.

---


### setGenerateRanking

```
void setGenerateRanking(boolean doRanking)
```

:   Sets whether or not ranking is to be performed.
    When a search method is capable of producing a ranked list
    of attributes, the user has the choice of seeing the results of a
    normal search or seeing a ranked list.

    :   **Parameters:**: `doRanking` - true if ranked list is to be produced

---


### getGenerateRanking

```
boolean getGenerateRanking()
```

:   Gets whether the user has opted to see a ranked list of
    attributes rather than the normal result of the search

    :   **Returns:**: true if a ranked list has been requested.


---


|  |  |  |
| --- | --- | --- |
| **Overview** \| **Package** \| **Class** \| **Tree** \| **Deprecated** \| **Index** \| **Help** \| **Weka's home** | |  |
| **PREV CLASS**   **NEXT CLASS** | **FRAMES**    **NO FRAMES**     **All Classes** |  |
| SUMMARY: NESTED \| FIELD \| CONSTR \| METHOD | DETAIL: FIELD \| CONSTR \| METHOD |  |


---
